# Cryptosporidiosis: A zoonotic disease concern

**DOI:** 10.14202/vetworld.2018.681-686

**Published:** 2018-05-23

**Authors:** Natapol Pumipuntu, Supawadee Piratae

**Affiliations:** One Health Research Unit, Faculty of Veterinary Sciences, Mahasarakham University, Maha Sarakham, Thailand

**Keywords:** cryptosporidiosis, diarrhea, waterborne disease, zoonosis

## Abstract

Cryptosporidiosis is considered to be a crucial zoonotic disease caused by worldwide distributing parasitic protozoa called *Cryptosporidium* spp. Cryptosporidiosis becomes a major public health and veterinary concern by affecting in human and various host range species of animals. Essentially, its importance of infection is increasing because of the high incidence in young children, immunocompromised persons, or immunodeficiency syndrome patients, especially in HIV/AIDS, and it is also one of the most causes of mortality in those patients who infected with *Cryptosporidium* spp. as well as young animals. All domestic animal, livestock, wildlife, and human can be potential reservoirs that contribute *Cryptosporidium* spp. to food and surface waters and transmitted to other hosts through fecal-oral route. The oocyst stage of *Cryptosporidium* spp. can remain infective and resistant to various environmental exposure and also resistant to many general disinfecting agents including chlorination which normally used in water treatment. Therefore, the understanding of these zoonotic pathogens is very essential in both animal and human health. This review focuses on the biology, life cycle, transmission, diagnosis, treatment, prevention, and control of this protozoan infection to emphasize and remind as the significant One Health problem.

## Introduction

Cryptosporidiosis is considered to be one of the most important diseases causing an intestinal infection to humans and animals [1]. The illness is primarily in mild diarrhea to severe in immune-compromised person, especially in HIV/AIDS, and patients who received immunosuppressive drugs. The etiological agent is the protozoan from *Cryptosporidium* genus [2] which has been reported that approximately 60% of them were the most common causative protozoan parasites of food and waterborne disease outbreak worldwide during 2004-2010 [3]. Human and animals get infected when they consume food and drink containing oocysts of these protozoa. Due to food and water transmission, incidence and prevalence of cryptosporidiosis are higher in less developed and developing countries where people are insufficient of basic infrastructure or fundamental facilities to avoid food and drinking water contaminated by infectious oocysts in feces [4]. In addition, the oocysts play a potential role to contribute *Cryptosporidium* spp. because they are tolerant to several chemicals and disinfectants including chlorine which is commonly used to treat in drinking water processing, also swimming pools, and water parks [5].

Twenty-three species and 61 valid genotypes of *Cryptosporidium* spp. have been described from a wide range of vertebrates including humans, mammals, wildlife, domestic livestock, reptile, birds, amphibians, and fish with causing asymptomatic or mild-to-severe gastrointestinal disease in its host species [6]. Several species have been isolated from immunocompromised humans such as *Cryptosporidium canis, Cryptosporidium felis, Cryptosporidium meleagridis, Cryptosporidium suis, Cryptosporidium muris*, and *Cryptosporidium andersoni* [7]. The most common three species of *Cryptosporidium* in human are *Cryptosporidium parvum, Cryptosporidium hominis*, and *C. meleagridis*; however, *C. parvum* and *C. hominis* are responsible for more than 90% of cryptosporidiosis cases [8]. Interestingly, almost 155 species of other mammals were reported as the non-human hosts of *C. parvum* [9] which indicated that the parasites are adapting and developing to infect many diverse hosts and able to be one of the major zoonotic diseases problems.

Cryptosporidiosis has been informed as a significant cause of diarrhea worldwide. In Southeast Asia, cryptosporidiosis is frequently reported in both humans and animals from many countries such as Laos, Vietnam, Philippines, Myanmar, Indonesia, Cambodia, Malaysia, and Thailand [10]. In Thailand, 6% of ocean water samples and 11% of river samples are contaminated with *Cryptosporidium* spp. [11]. Moreover, *C. muris, C. parvum, C. meleagridis, C. hominis, C. felis*, and *C. canis* have been isolated from HIV/AIDS cases in Bangkok [12] with the prevalence at 19-34% from 1996 to 2009 [13]. In other regions, cryptosporidiosis is also recognized as a major concern by the increasing cases of immune-compromised individuals and children in many countries of the Americas such as Costa Rica, Argentina, Brazil, and the USA while the incidence of cryptosporidiosis in Europe is increasing by climate changes as heavy rainfall or flood and this pathogen can contaminate in drinking water. Therefore, the reports of cryptosporidiosis cases in Europe are higher than previous years, and *C. hominis* was identified as the most common pathogen [1]. *Cryptosporidium* spp. is associated with severe diarrhea, mortality increasing, and negative impact of development and growth to the children in Africa. More than that, HIV/AIDS outbreak and malnutrition status of some developing countries in Africa can contribute the high prevalence of the contagion in this region [14].

Cryptosporidiosis in livestock is becoming the significant problems for animal health (both subclinical and clinical) and economic losses [15] because of increasing veterinary services and labor costs, increasing animal health-care cost, and decreasing a growth rate of animals and mortality of severe animals. Previous reports of cryptosporidiosis in livestock in Thailand were 31.5%, 5.7%, and 8.7% in dairy farms, individual animals, and dairy herd, respectively [16]. Moreover, 30% of buffalo farms were infected with *Cryptosporidium* spp., and *C. parvum* was the most species contributed in livestock animal [17]. For other animals, the prevalence of cryptosporidiosis showed 2.1% in dog and 2.5% in cat. For wildlife animal such as in long-tailed macaques which live closely and normally contact to human communities in Thailand, it showed a prevalence about 1% but pose an important human health risk because infection in this animal can be occurred although less number of oocysts [18]. Here, we review the general information of *Cryptosporidium* spp. which researches are needed for understanding the biology, life cycle, transmission, diagnosis, treatment, prevention, and control of these protozoa.

## Morphology and Life Cycle of *Cryptosporidium* Species

**Figure-1:**
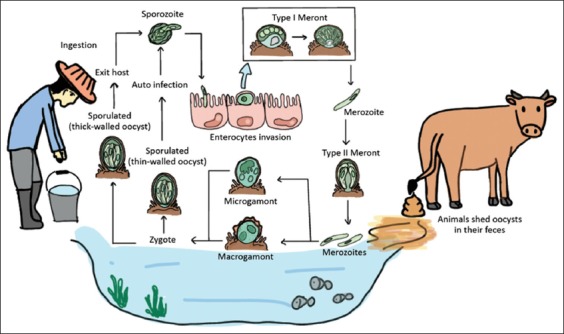
Life cycle and transmission of *Cryptosporidium* spp. (picture by Thamonphan Wimonsrinarachai).

*Cryptosporidium* has a complex life cycle involving both sexual (meiosis) and asexual replication (mitosis) but monoxenous cycle [19]. Furthermore, it has many morphology formations to complete in its life cycle. Oocysts are excreted in the environment from human and animals through the feces. After the host ingests infective oocyst, excystation will occur to release four sporozoites. The sporozoites will invade the gastrointestinal tract and develop to merozoites, gamonts, and oocysts. Oocysts exist in two forms: Thin wall will reinfect in the gastrointestinal tract and thick wall will excrete in the environment through feces which shows in Figure-1. A detailed account of the life cycle starts from sporulated oocyst (which is rarely morphometric differences among different species) released by the infected host. After that, vertebrate host ingests sporulated oocyst through consumption of contaminated food or drink, and the process of excystation will occur to release 4 infectious sporozoites. This progression occurs in the gastrointestinal tract triggered by CO_2_, the temperature of 37°C, pancreatic enzymes, and pH acidic of bile salts [20].

Oocyst has two types, one is the thin wall (one layer of a protein-lipid–carbohydrate matrix), and another is the thick wall (consisting of inner and outer oocyst walls). Only the thick wall is shedding into the environment. The size of *Cryptosporidium* oocysts typical have nearly spherical with small size around 4-6 µm on the average and have obscure internal structures. The hallmark characteristic of mature oocysts contains four sporozoites but no sporocyst.

The sporozoites are nucleated, spindle shape with 5.0 µm×0.5 µm. The apical complex part plays function in the gliding motility to access the target cell. The nucleus is on the center of the cell. Sporozoites recognize and penetrate to the target host cells including stomach (*C. muris* and *C. andersoni*) and intestine (such as *C. parvum* and *C. hominis*) [7]. Invaded sporozoites are from parasitophorous vacuole surroundings where they can differentiate into trophozoite stage (1.5-2.5 μm in diameter) within the parasitophorous vacuole.

The trophozoites commence three mitosis divisions (merogony) to produce type I meronts which contain 8 merozoites (morphologically similar to sporozoites which are rod-like 0.4 μm×1.0 μm) [21]. Type I merozoites can invade host cell again and reproduce asexual to from either type I meronts which contains eight merozoites or type II meronts which contain only four merozoites. Though, merozoites release from type II meronts are less uniform in shape, slightly larger and less active when compared to merozoites release from type I meronts.

Type II merozoites can be developed to microgamont (male) or macrogamont (female) which undergoes sexual reproduction (gametogony). Each microgamont produces sixteen microgametes which are rod-like, non-flagellated and 1.4 μm×0.5 μm in size. It will fertilize with a unicellular adjacent macrogamont, a spherical to an oval structure of 4 to 6 μm in diameter with a large central nucleus [22]. After two mitosis divisions, zygote develops either a thin-walled oocyst covering only a single layer membrane or a thick-walled oocyst containing two-layered membranes. The thick-walled oocysts are released through feces and tolerance for months in the unsuitable environment, whereas the thin-walled oocysts can cause reinfection within the gastrointestinal tract of the same host by rupturing and releasing infective sporozoites [23].

## Transmission

Transmission routes of this parasite can be divided into two methods, direct and indirect transmissions. Direct transmission occurs through a fecal-oral route by accidental ingestion, and the *Cryptosporidiu*m oocytes excreted from feces. The transmission happens between animal to animal, animal to human (zoonosis), or vice versa, from human to animal (anthroponotic or reverse zoonosis) [24], and human-to-human transmissions [25] which usually emerges in swimming pools, water parks, day care center, hospital, and during anal sexual contact with human feces. In addition, this process is frequently raised by sexual intercourse behavior of men who have sex with men through fecal-oral route [26]. Moreover, the direct transmission can occur through direct exposure to infected animals such as contact with infected calves by veterinarians or animal researchers who have a high risk to contact with the infected animals.

For indirect transmission, this protocol can occur by cross-contaminated in foodstuff, food materials, drinking water, and many fomites such as clothes and footwear used in livestock farm or wildlife park which have been exposed with the feces of an infected human or animal. It can infect and live in epithelial surfaces of the intestine in humans included wide range of vertebrate animals and passed from feces or stool, then, contaminated in the environment (environmental contamination) such as soil and water sources; pond, river, wastewater, sewage or slurry, even many water containers especially insufficiently treated public water supplies. The transmission and distribution are presented following a high rate of rainfall and flooding event [27]. Thus, the infection of human and animal usually commences with the ingestion of oocysts that contain four sporozoites within.

## Diagnosis

Standard methods of detection are oocyst examination by the microscopic method. However, *Cryptosporidium* has been detected by various approaches based on each laboratory systems which consequently differ in examination standard. Microscopic examination is the conventional method for the detection of oocyst in stool samples which can perform by wet mount followed by staining with a special dye such as acid-fast dye, fluorescence, or immunofluorescence to enhance the sensitivity of detection [28]. Acid-fast stained oocysts are intermittently red with size around 4-6 µm and contain crescent-shaped sporozoites. The acid-fast dye familiar used are the modified Ziehl–Neelsen technique, modified dimethyl sulfoxide, safranin-methylene blue, and modified Koster [29,30]. Fluorochrome stains are increasing sensitivity but sometimes stained non-specific of oocyst-like structures in fecal debris [31]. In addition, staining after concentration methods which concentrate both quantities of feces and oocysts can enhance the opportunity of detection and can do with fresh, frozen, and preserved stool [32]. Prominent fecal concentration techniques are executed by sedimentation using formalin-ethyl acetate and floatation such as modified zinc sulfate, the Sheather’s sugar flotation, and saturated sodium chloride.

Nevertheless, microscopic examination is labor and time-consuming and lacks sensitivity and specificity, and misdiagnosis is also frequently occurred as insufficient knowledge of the oocyst morphology and biological characteristics. Consequently, immunological-based techniques have been developed as alternatives ways of detections including direct fluorescent antibody, enzyme immunoassay, enzyme-linked immunosorbent assay (ELISA), indirect ELISA, and dipstick-like tests [33].

Although immunological-based techniques increase laboratory efficiency by more sensitivity and specificity, reducing labor, time, and costs, many oocyst antigens are conserved within the genus of *Cryptosporidium* and appear in several species. There are no antibodies to differentiate *Cryptosporidium* species reliably. DNA-based detection methods have been used to identify *Cryptosporidium* species which include polymerase chain reaction (PCR) assay and DNA sequencing of PCR products [34], PCR restriction fragment length polymorphism [35,36], real-time PCR assays, nested PCR [37], multiplex PCR [38], and other modified PCR. Several genetic regions such as the 18s rDNA, *Cryptosporidium* oocyst wall protein, heat shock protein 70, Acetyl-CoA gene, b-tubulin gene, Cp15 gene, Cp 11 gene, dihydrofolate reductase inhibitors (*dhfr*), microsatellite loci, and 60 kDa glycoprotein (*gp60*) have been commonly used to identify *Cryptosporidium* isolates in animal and human infections [33,39]. Moreover, detection of cryptosporidiosis can be done with intestinal biopsy [40]. Infection part of the intestine can be stained with hematoxylin and eosin which will demonstrate parasites in each stage attached to the epithelial cells.

## Treatment

According to the previous information, most patients and infected animals which have vigorous immune response can convalesce themselves without any treatments. Some clinical symptoms including watery fever, vomiting, nausea, stomach cramp, and dehydration can be managed by some supportive treatments as fluid and electrolyte replacement, anti-nausea drugs, antiemetic drugs, or analgesic drugs. Those medications can relieve the symptoms from cryptosporidiosis, but an antiprotozoal therapy is necessary in some cases.

Nitazoxanide is the most effective drugs for treating patients who infected with *Cryptosporidium* spp., and it is the only anti-cryptosporidial agent which has been approved for the treatment of cryptosporidiosis in humans by the US Food and Drug Administration. However, it is not commercially available or not yet widely used at the present time [1]. In addition, nitazoxanide cannot effective without a good immune response of the host so that it cannot be used effectively in immunocompromised patients [41]. For animals, they have few reports that studied the effect of nitazoxanide against clinical infections of *Cryptosporidium* in animals which demonstrated that nitazoxanide could decrease *Cryptosporidium* oocysts excretion [42,43]. Notwithstanding, it is also not yet generally used in animals.

## Prevention

*Cryptosporidium* spp. can transmit and infect susceptible hosts by a major route of fecal to oral as cross-contamination in raw food and water from reservoir animals in community, farms, or abattoirs and some mechanical vectors such as cockroaches and flies [44]. Then, humans can get infected by their oocysts by ingesting this contaminated food and water with unprocessed or improper hygiene. Therefore, in a case of human prevention, the best strategy to prevent transmission of *Cryptosporidium* spp. is a practice of good personal hygiene including handwashing before preparing and consuming food, after using a toilet, and after contacting with diarrhea patients, children, and some animals or livestock. Raw food and water must be suitably cleaned, washed, heated, cooked, or boiled before consumption and drinking, respectively. More than that, patients who have diarrhea symptom should realize not to swim in a public swimming pool, public water park, or river for preventing a transmission to others and people who swim in a swimming pool, water park, or river should recognize a potential risk of disease infection if they swallow the water [45].

Preventive measures that diminish transmission of *Cryptosporidium* spp. among animals, especially in livestock, should be emphasized on a primary prevention which reduce or eliminate causative risk factors by limiting the amount of animals density in the farms or stocks, minimizing a contraction between personnel, calves, and other herds, keeping young animals or susceptible hosts that have high risk of infection separated from adult animals, and keeping a short calving period of animals which may decrease the opportunities for *Cryptosporidium* spp. to spread within animal herds [46]. In human living areas, livestock husbandry, and their drinking water, the destruction of *Cryptosporidium* oocysts can be managed by heat or chemical disinfection such as hydrogen peroxide, sterilization processes using steam, ethylene oxide, chlorine dioxide, ozone (O_3_), and ultraviolet light (UV light) which have been used to sterilize drinking water, but some of the disinfectants are not commonly practical procedure. However, all of the chemical disinfection can be used to prevent and control the occurrence of cryptosporidiosis and to reduce mortality and morbidity rate in both infected in both human and animals [47,48].

## Conclusion

*Cryptosporidium* spp. distributes worldwide, especially in undeveloped and developing countries. The distribution has arisen when *Cryptosporidium*’s oocyst is defecated to water surface from human and animals (wildlife, domestic animals, and livestock) through the feces. The excreted oocysts are sustainable and tolerance with disinfectant, dilute bleach, and chlorine. Transmission occurs when hosts expose with *Cryptosporidium*’s oocyst mainly by fecal-oral route through contaminated food and water or contact directly with animal feces and indirectly by cross contamination. Laboratory diagnosis of *Cryptosporidium* infection normally uses oocyst staining and examines under microscopy. However, these methods cannot differentiate morphology of each *Cryptosporidium* species, so immunological methods and molecular techniques are playing more role for identification to species. Most of robust infected human and animals are asymptomatic and mild diarrhea, but violent symptoms will occur in the immunocompromised host. The best strategy to prevent and narrow down the spreading of this disease is keeping good personal hygiene in human coupled with reduction, control, or elimination the causative risk factors for other animals.

## Authors’ Contributions

NP and SP conceived the data, wrote the manuscript, reviewed and edited the manuscript. Both authors read and approved the final manuscript.

## References

[1] Bamaiyi P, Redhuan N (2017). Prevalence and risk factors for cryptosporidiosis:A global, emerging, neglected zoonosis. Asian Biomed.

[2] Karanis P, Kourenti C, Smith H (2007). Waterborne transmission of protozoan parasites:A worldwide review of outbreaks and lesson learnt. J. Water Health.

[3] Baldursson S, Karanis P (2011). Waterborne transmission of protozoan parasites:Review of worldwide outbreaks–An update 2004–2010. Water Res.

[4] Burnet J.B, Penny C, Ogorzaly L, Cauchie H.M (2014). Spatial and temporal distribution of*Cryptosporidium*and*Giardia*in a drinking water resource:Implications for monitoring and risk assessment. Sci. Total Environ.

[5] Fayer R, Trout J.M, Xiao L, Morgan U.M, Lal A.A, Dubey J.P (2001). *Cryptosporidium*
*canis*n. sp. from domestic dogs. J. Parasitol.

[6] Ryan U, Fayer R, Xiao L (2014). *Cryptosporidium*species in humans and animals:current understanding and research needs. Parasitology.

[7] Fayer R (2010). Taxonomy and species delimitation in*Cryptosporidium*. Exp. Parasitol.

[8] Xiao L, Ryan U.M (2004). Cryptosporidiosis:An update in molecular epidemiology. Curr. Opin. Infect. Dis.

[9] Šlapeta J (2013). Cryptosporidiosis and*Cryptosporidium*species in animals and humans:a thirty colour rainbow?. Int. J. Parasitol.

[10] Lim Y.A, Mahdy M.A, Surin J (2013). Unravelling*Cryptosporidium*and*Giardia*in Southeast Asia. Parasites and their Vectors. Southeast Asia*Cryptosporidium*and*Giardia*have been reported in countries such as Cambodia, Indonesia.

[11] Koompapong K, Sukthana Y (2012). Seasonal variation and potential sources of*Cryptosporidium*contamination in surface waters of Chao Phraya River and Bang Pu nature reserve pier, Thailand. Southeast Asian J. Trop. Med. Public Health.

[12] Srisuphanunt M, Saksirisampant W, Karanis P (2011). Prevalence and genotyping of*Cryptosporidium*isolated from HIV/AIDS patients in urban areas of Thailand. Ann. Trop. Med. Parasitol.

[13] Berger S (2017). Infectious Diseases of Thailand:2017 Edition.

[14] Squire S.A, Ryan U (2017). *Cryptosporidium*and*Giardia*in Africa:Current and future challenges. Parasit. Vectors.

[15] Santin M (2013). Clinical and subclinical infections with*Cryptosporidium*in animals. N Z Vet J.

[16] Jittapalapong S, Pinyopanuwat N, Chimnoi W, Siripanth C, Stich R.W (2006). Prevalence of*Cryptosporidium*among dairy cows in Thailand. Ann NY Acad Sci.

[17] Inpankaew T, Jiyipong T, Wongpanit K, Pinyopanuwat N, Chimnoi W, Kengradomkij C, Xuan X, Igarashi I, Xiao L, Jittapalapong S (2014). Molecular detection of*Cryptosporidium*spp. infections in water buffaloes from northeast Thailand. Trop. Anim. Health Prod.

[18] Sricharern W, Inpankaew T, Keawmongkol S, Supanam J, Stich R.W, Jittapalapong S (2016). Molecular detection and prevalence of*Giardia duodenalis*and*Cryptosporidium*spp. among long-tailed macaques (*Macaca fascicularis*) in Thailand. Infect. Genet. Evol.

[19] Bouzid M, Hunter P.R, Chalmers R.M, Tyler K.M (2013). *Cryptosporidium*pathogenicity and virulence. Clin. Microbiol. Rev.

[20] O'Donoghue P.J (1995). *Cryptosporidium*and cryptosporidiosis in man and animals. Int. J. Parasitol.

[21] Cacciò S.M, Widmer G (2013). *Cryptosporidium*:Parasite and Disease.

[22] Current W, Reese N.C (1986). A comparison of endogenous development of three isolates of*Cryptosporidium*in suckling mice. J. Protozool.

[23] Tzipori S, Ward H (2002). Cryptosporidiosis:Biology, pathogenesis and disease. Microbes. Infect.

[24] Hubalek Z (2003). Emerging human infectious diseases:Anthroponoses, zoonoses, and sapronoses. Emerg. Infect. Dis.

[25] Xiao L, Feng Y (2008). Zoonotic cryptosporidiosis. FEMS Immunol. Med. Microbiol.

[26] Pedersen C, Danner S, Lazzarin A, Glauser M.P, Weber R, Katlama C, Barton S.E, Lundgren J.D (1996). Epidemiology of cryptosporidiosis among European AIDS patients. Sex Transm. Infect.

[27] Jiang J, Alderisio K.A, Xiao L (2005). Distribution of*Cryptosporidium*genotypes in storm event water samples from three watersheds in New York. Appl. Environ. Microbiol.

[28] Alles A.J, Waldron M.A, Sierra L.S, Mattia A.R (1995). Prospective comparison of direct immunofluorescence and conventional staining methods for detection of*Giardia*and*Cryptosporidium*spp. in human fecal specimens. J. Clin. Microbiol.

[29] Chalmers R.M, Katzer F (2013). Looking for*Cryptosporidium*:The application of advances in detection and diagnosis. Trends Parasitol.

[30] Koonakosit R, Sriurairatna S, Petchclai B (1992). Microscopic examination of*Cryptosporidium*oocysts in diarrhoeal stools. J. Med. Assoc. Thai.

[31] Campbell A.T, Haggart R, Robertson L.J, Smith H.V (1992). Fluorescent imaging of*Cryptosporidium*using a cooled charge couple device (CCD). J. Microbiol. Methods.

[32] Pacheco F.T, Silva R.K, Martins A.S, Oliveira R.R, Alcântara-Neves N.M, Silva M.P, Soares N.M, Teixeira M.C (2013). Differences in the detection of*Cryptosporidium*and*Isospora (Cystoisospora)*oocysts according to the fecal concentration or staining method used in a clinical laboratory. J. Parasitol.

[33] Fayer R, Morgan U, Upton S.J (2000). Epidemiology of*Cryptosporidium*:Transmission, detection and identification. Int. J. Parasitol.

[34] Simonato G, Frangipane di Regalbono A, Cassini R, Traversa D, Tessarin C, Di Cesare A, Pietrobelli M (2017). Molecular detection of*Giardia duodenalis*and*Cryptosporidium*spp. in canine faecal samples contaminating public areas in Northern Italy. Parasitol. Res.

[35] Xiao L, Escalante L, Yang C, Sulaiman I, Escalante A.A, Montali R.J, Fayer R, Lal A.A (1999). Phylogenetic analysis of*Cryptosporidium*parasites based on the small subunit rRNA gene locus. Appl. Environ. Microbiol.

[36] Xiao L, Morgan U.M, Limor J, Escalante A, Arrowood M, Shulaw W, Thompson R.C, Fayer R, Lal A.A (1999). Genetic diversity within*Cryptosporidium parvum*and related*Cryptosporidium*species. Appl. Environ. Microbiol.

[37] Abdelsalam I.M, Sarhan R.M, Hanafy M.A (2017). The impact of different copro-preservation conditions on molecular detection of*Cryptosporidium*species. Iran J. Parasitol.

[38] Mero S, Kirveskari J, Antikainen J, Ursing J, Rombo L, Kofoed P.E, Kantele A (2017). Multiplex PCR detection of*Cryptosporidium*sp*Giardia lamblia*and*Entamoeba histolytica*directly from dried stool samples from Guinea-Bissauan children with diarrhoea. Infect. Dis. (Lond).

[39] Khan A, Shaik J.S, Grigg M.E (2017). Genomics and molecular epidemiology of*Cryptosporidium*species. Acta Trop.

[40] Doganci T, Araz E, Ensari A, Tanyuksel M, Doganci L (2002). Detection of*Cryptosporidium parvum*infection in childhood using various techniques. Med. Sci. Monit.

[41] Gargala G (2008). Drug treatment and novel drug target against*Cryptosporidium*. Parasite.

[42] Viel H, Rocques H, Martin J, Chartier C (2007). Efficacy of nitazoxanide against experimental cryptosporidiosis in goat neonates. Parasitol. Res.

[43] Ollivett T.L, Nydam D.V, Bowman D.D, Zambriski J.A, Bellosa M.L, Linden T.C, Divers T.J (2009). Effect of nita-zoxanide on cryptosporidiosis in experimentally infected neonatal dairy calves. J. Dairy Sci.

[44] Conn D.B, Weaver J, Tamang L, Graczyk T.K (2007). Synanthropic flies as vectors of*Cryptosporidium*and*Giardia*among livestock and wildlife in a multispecies agricultural complex. Vector Borne Zoonotic Dis.

[45] Rossle N.F, Latif B (2013). Cryptosporidiosis as threatening health problem:A review. Asian Pac. J. Trop. Biomed.

[46] Hoar B.R, Atwill E.R, Elmi C, Farver T.B (2001). An examination of risk factors associated with beef cattle shedding pathogens of potential zoonotic concern. Epidemiol. Infect.

[47] De Graaf D.C, Vanopdenbosch E, Ortega-Mora L.M, Abbassi H, Peeters J.E (1999). A review of the importance of cryptosporidiosis in farm animals. Int. J. Parasitol.

[48] Ghazy A.A, Abdel-Shafy S, Shaapan R.M (2016). Cryptosporidiosis in animals and man:3. Prevention and control. Asian J. Epidemiol.

